# Impacts of the COVID-19 Pandemic on Anxiety and Depressive Symptoms in Pregnant Women and Related Perinatal Outcomes

**DOI:** 10.3390/jpm13010094

**Published:** 2022-12-30

**Authors:** Huan Han, Luyao Wang, Wenjing Lu, Jiaqi Dong, Yinuo Dong, Hao Ying

**Affiliations:** 1 Department of Obstetrics, Shanghai First Maternity and Infant Hospital, Tongji University School of Medicine, Shanghai 201204, China; 2 Shanghai Key Laboratory of Maternal Fetal Medicine, Shanghai 200092, China

**Keywords:** COVID-19, pregnant women, anxiety symptoms, depression symptoms, pregnancy outcomes

## Abstract

To evaluate the impacts of the COVID-19 pandemic on anxiety and depression symptoms in pregnant women and their relationship with pregnancy outcomes, 1087 pregnant women completed online questionnaires. Anxiety symptoms were measured using the Self-Rating Anxiety Scale (SAS). Depression was assessed using the Edinburgh Postnatal Depression Scale (EPDS), and the Pittsburgh Sleep Quality Index (PSQI) was used to assess sleep quality. Univariate analysis and logistic regression analysis were used to determine the association between depression and anxiety symptoms, participants’ characteristics, and pregnancy outcomes. Of the 986 pregnant women who were included in this study, the rates of anxiety symptoms and depressive tendencies were 13.4% and 18.3%, respectively. Sleep disorder ((Adjusted odds ratio, AOR = 4.166; 95% confidence interval, CI: 2.797–6.205), time spent paying attention to the epidemic per day (≥1 h/d AOR = 1.568; 95% CI: 1.052–2.338), and the time spent with their spouses (Increase AOR = 0.629; 95% CI: 0.409–0.967) were associated with the risk of anxiety. Sleep disorder (AOR = 3.839; 95% CI: 2.718–5.432) and educational level (bachelor’s degree or above AOR = 1.833; 95% CI: 1.004–3.345) were associated with the risk of depression. Psychological status was not correlated with the pregnancy outcomes (*p* > 0.05). Anxiety and depression symptoms were common among pregnant women during the COVID-19 pandemic. Special attention should be paid to manage their risk factors.

## 1. Introduction

Since the end of 2019, the coronavirus disease 2019 (COVID-19) has been spreading rapidly. As a global pandemic recognized by the World Health Organization (WHO) on 11 March 2020 [1], it poses serious threats to public health. A recent study showed that multifaceted public health interventions are temporarily associated with improved control over the COVID-19 pandemic [2]. However, due to the rapid spread, strong infectivity, and uncertain treatment of the virus, together with consequential social restrictions such as quarantines and lockdowns, the epidemic has affected the psychological health of the public worldwide [3]. Psychological disorders are most likely affecting the more vulnerable populations, such as pregnant women and college students [4,5].

Pregnant women are undergoing subtle changes both mentally and physically, and as a result of the aggravation of body load and the changes in hormone levels, they are prone to various adverse emotions during pregnancy, especially during pandemics with uncertain endpoints [6]. Previous studies have shown that psychological problems in pregnant women have significantly increased since the COVID-19 outbreak [7], with anxiety and depression being the most common ones during COVID-19 [8,9]. Recent studies from different countries and regions have shown that the incidence of depression and anxiety varies significantly. According to one study, 29.6% of pregnant women in China present with depressive symptoms [10]. In Spain, the prevalence of mental health issues was reported as 58% [11]. Anxiety symptoms reportedly had a prevalence of 51% in Spain [11] and 68% in Italy [12]. Mental disorders during pregnancy are associated with adverse maternal and neonatal outcomes, including spontaneous abortion, preterm birth, low birth weight, bleeding, preeclampsia, and cesarean delivery [13,14,15].

Although previous studies have shown that the psychological impact of a major disaster has a wider and longer effect on people than physical injuries, mental health still attracts fewer public and medical resources [16]. To better guide clinical practice, we conducted this study to assess the psychological problems of pregnant women in Shanghai during the COVID-19 pandemic and their relationship with pregnancy outcomes.

## 2. Materials and Methods

### 2.1. Study Participants

This study was performed at the Shanghai First Maternity and Infant Hospital, Tongji University School of Medicine, between 26 February and 26 March 2020. All of the women who were confirmed to be pregnant by ultrasonography and registered to attend antenatal care at the Department of Obstetrics were required to complete online psychological questionnaires through a platform named sojump, regardless of gestational age. The pregnancy outcomes of these pregnant women who completed the questionnaires were inquired about through telephone interviews or hospitalization data, including gestational age at delivery, postpartum hemorrhage and birth weight. Patients were excluded if (1) their age was <18 years; (2) they could not comprehend or complete the questionnaires independently; (3) they had a definite diagnosis of psychiatric disease before; or (4) they terminated pregnancy due to severe fetal malformations. A total of 1087 participants received the questionnaires. A total of 101 patients were lost to follow-up; thus, 986 pregnant women remained for the analysis. This study was approved by the Ethics Committee (KS21409), and written informed consent was obtained from each patient.

### 2.2. Candidate Risk Indicators

The online survey contained three parts: The first part contained questions on sociodemographic and clinical characteristics such as age, educational level, annual household income, employment status, residence, gravidity, parity, number of fetuses, gestational week, pregnancy complications, and so on. The second part included epidemic-related questions related to degree of epidemic concern, time spent with a spouse, and whether they were quarantined for 2 weeks. The third part was composed of three questionnaires: Self-Rating Anxiety Scale (SAS), Edinburgh Postnatal Depression Scale (EPDS), and Pittsburgh Sleep Quality Index (PSQI) for evaluating psychological status.

### 2.3. Questionnaires

SAS and PQSI are the most widely used questionnaires for anxiety and sleep disorders in our clinical work, while EPDS is specifically for the perinatal population. In addition, considering their clear items, short completion time, good patient cooperation and high reliability and validity proved by previous studies [17,18,19], these scales were selected in this study.

The Self-Rating Anxiety Scale (SAS) was compiled by Zung in 1971 [20] and was introduced in China in the 1980s. It has been widely used to assess adult anxiety and has demonstrated adequate validity and acceptable reliability. The SAS includes 20 items using 4-point response options ranging from 1 (never) to 4 (very often) to capture symptoms of antenatal anxiety. The responses are used to give a total score that can range from 20 to 80, and then the standard score equal to the integral part of the figure of total score is multiplied by 1.25. A standard score ≥50 is recommended to identify women with antenatal anxiety, with higher scores indicating higher levels. A score of <50 indicates no anxiety, 50–59 points mild anxiety, 60–69 points moderate anxiety, and more than 69 points severe anxiety.

The Edinburgh Postnatal Depression Scale (EPDS) was developed by the Livingston and Edinburgh Health Center in the United Kingdom in 1987 to screen depressive emotions in pregnant and postpartum women [21]. The scale has a total of 10 questions, with each question having a score of 0 to 3. The higher the score, the higher the degree of depression. In this study, 10 and 12 were used as the cutoff points, with <10 indicating a low risk of prenatal depression, 10–12 a moderate risk of prenatal depression, and ≥13 a high risk of prenatal depression.

The Pittsburgh Sleep Quality Index (PSQI) was used to measure the subjective sleep quality of the participants in the last month [22]. This comprised seven factors: subjective sleep quality, sleep latency, sleep duration, sleep efficiency, sleep disturbance, sleep medication use, and daytime dysfunction. The total score (ranging from 0 to 21) was obtained from the sum of the seven factors. A PSQI score of 5 was used as the cut-off value, and a PSQI score of more than 5 indicated poor sleep quality.

### 2.4. Statistical Methods

SPSS software (version 21.0) was used for the data analysis. Continuous variables were presented as the mean ± standard deviation (SD) if the data distribution was normal and as the median and quartiles if the data distribution was skewed or nonnormal. Comparisons of continuous variables between two groups were performed using the t test or Mann–Whitney U test depending on the distribution of the samples. Categorical variables were presented as frequencies and percentages. Chi-square (χ^2^) tests were used for comparisons of categorical variables between two groups of categorical variables. Fisher’s exact test was used when an expected-value problem occurred. Taking the presence of anxiety and depressive symptoms as dependent variables, and demographic characteristics, pregnancy and epidemic-related factors, and sleep status as independent variables, logistic regression was used to analyze the risk factors affecting the psychological status of pregnant women during the pandemic. Statistical significance was set at *p* < 0.05.

## 3. Results

A total of 1087 participants received the questionnaires; however, 101 patients were lost to follow-up. Thus, 986 pregnant women were included in the analysis, and none of them were infected during the pandemic. The distribution of the psychological symptoms is presented in Table 1. The presence of symptoms associated with anxiety and depression was reported by 13.4% and 18.3% of the pregnant women, respectively, comprising mainly mild to moderate symptoms. About 36.7% of the pregnant women had different degrees of sleep disorders.

The sociodemographic and clinical characteristics and epidemic-related factors are presented in Table 2. The prevalence of anxiety symptoms during the outbreak period was not associated with the general characteristics of pregnant women, such as age, residence, income, education, and gestational week, or pregnancy complications, including hypertension, fetal growth restriction (FGR) and abnormal amniotic fluid (*p* > 0.05). However, it was related to epidemic-related factors, including the time spent paying attention to the epidemic every day and time spent with their spouses. Pregnant women who were concerned about the epidemic for less than 1 h a day or increased the time spent with their spouses were less likely to have anxiety symptoms (*p* < 0.05). The time pregnant women spent with their spouses during the pandemic and gestational age affected the risk of depression symptoms (*p* < 0.05). Pregnant women who increased their time with their spouses were less likely to have a moderate to high risk of depression. There was no significant correlation between pregnancy-related complications and the incidence of depression (*p* > 0.05). In addition, our study showed that the occurrence of sleep disorders was significantly associated with both anxiety and depression symptoms (*p* = 0.000).

The results of the logistic regression analysis (Table 3) showed that sleep disorder was a common risk factor for antenatal anxiety and depression symptoms during the COVID-19 pandemic. Pregnant women with sleep disorders were 4.166 and 3.839 times more likely to develop anxiety and depression symptoms, respectively, than those without sleep disorders. Additionally, the time spent paying attention to the epidemic and the time spent with their spouses were associated with the risk of anxiety, while education level was associated with the risk of depression. The probability of anxiety symptoms in pregnant women who spent ≥1 h per day on pandemic information was 1.568 times that of women who spent <1 h. Increased time with their spouses was a protective factor for the occurrence of anxiety symptoms (OR 0.629, 95% CI: 0.409–0.967). The risk of depressive symptoms in pregnant women with a bachelor’s degree or above was 1.833 times that in those with a high school degree or below.

Among the 986 pregnant women who were followed up in this study, the incidence of preterm birth, postpartum hemorrhage and macrosomia was 4.0%, 2.1%, and 3.6%, respectively. During the COVID-19 pandemic, the presence of anxiety, depressive symptoms or sleep disorders in pregnant women was not significantly correlated with pregnancy outcome (*p* > 0.05) (Table 4).

## 4. Discussion

As a result of the rapid spread, high mortality and morbidity associated with COVID-19, governments worldwide implemented various measures to avoid further spread of the pandemic and reduce the number of new infections. We conducted this study from 26 February to 26 March 2020, the initial stage of the pandemic. In this period, although Wuhan was the city most seriously affected by the pandemic, people in Shanghai were also affected due to the separation from family members, the loss of freedom and uncertainty over the epidemic, and all other uncomfortable experiences. During the outbreak of COVID-19, pregnant women often faced more stresses than other adults. In addition to worrying about being infected themselves, they were also concerned about the safety of the fetus and long-term prognosis. We found that 13.4% and 18.3% of participants had symptoms of anxiety and depression, respectively, due to the COVID-19 pandemic. The prevalence of anxiety and depression in pregnant women was 10.2% and 9.3%, respectively, before the pandemic in Shanghai [23]. Although quite a few studies have reported that the risk of anxiety and depression in pregnant women has significantly increased during the pandemic [7,8,9], the guidelines for psychological crisis intervention specifically for COVID-19, which were published by the National Health Commission of China, have still not categorized perinatal women as a vulnerable population.

Our study investigated the demographic characteristics that likely affected pregnant women’s psychological health during the pandemic. The results indicated that educational level was an important factor related to the development of depression during pregnancy—the higher the educational level, the higher the risk of depression symptoms. These results differ from previous studies conducted in other parts of China and other countries [24,25,26]. The difference may be related to the fact that women with high educational levels were more sensitive to the pandemic. At its beginning, information about the pandemic was mostly vague, and unknown treatment methods and development trends may have made them more nervous than women with lower educational levels.

Previous studies have revealed that the employment status of pregnant women is closely related to their psychological status [27,28]. A recent study found that approximately 35% of pregnant women in India had job-related concerns [29]. Loss of employment and reduced salaries during the pandemic increased economic pressures and financial concerns of the family, thereby increasing the risk of mental disorders. Our study showed that there was no correlation between the employment status of pregnant women and their psychological status, which may be attributable to the active medical policy promoted by the Chinese government. This policy exempted pandemic-related costs, including screening, diagnosis, and treatment of COVID-19.

COVID-19 related lifestyle changes affect anxiety and depression. In this study, we found that time spent paying attention to the pandemic, time spent with their spouse, and sleep quality were all important factors associated with mental health during pregnancy. Logistic regression analysis results showed that sleep disorders were a common risk factor for anxiety and depression. During the COVID-19 pandemic, measures taken to control the virus, such as quarantine, have greatly changed people’s daily behavior, which may be accompanied by poor eating habits, sedentary lifestyle, and reduced physical activity, thereby leading to sleep disorders [30]. Previous studies have shown that lifestyle changes can improve sleep patterns, thus reducing subsequent mental health risks [31]. The WHO guidelines on physical activity and sedentary behavior provide recommendations for regular strength training for pregnant women [32]. Previous studies have indicated that regular activity during pregnancy can reduce the likelihood of anxiety and depression [33]. Diet has also been proven to be related to mental health management. It has been proven that students who experience negative emotions tend to consume more high-calorie and junk foods, while those who feel positive tend to consume more fruits and vegetables [34]. A recent study revealed that compared with maternal dietary consumption before the pandemic, the consumption of vegetables, fruits, and dairy products decreased significantly during the pandemic [35]. Unfortunately, our research with limited data failed to confirm diet- or exercise-related results. However, it is still worth noting that during the pandemic, obstetricians should pay more attention to the sleep status of pregnant women, guide their exercise, and improve their eating habits and sleep quality to reduce the risk of anxiety and depression symptoms.

The time pregnant women spent thinking about the pandemic was positively correlated with the risk of anxiety symptoms; people who spent ≥1 h on pandemic information per day were more likely to have anxiety symptoms. In the early stage of the pandemic, a massive amount of unverified information, insufficient understanding of the disease, tense public opinion, and material shortages in some areas transmitted more uncertainty to pregnant women, resulting in anxiety symptoms. People who pay less attention to pandemic information may be less sensitive to its severity, resulting in a relatively lower incidence of anxiety.

Active family support can effectively promote positive emotions and reduce discomfort such as prenatal anxiety and depression. During the outbreak period, although lockdown was not an uncomfortable experience, family members spent more time together than ever before, which increased the interaction and communication between pregnant women and their partners. Pregnant women may receive more support from their spouses who play an important role in preventing and alleviating the occurrence and development of mental disorders during pregnancy. Our results showed that pregnant women who spent more time with their partners during the pandemic had a lower risk of developing anxiety symptoms, which was consistent with the results of previous studies [36,37,38].

It is unclear whether the psychological status of pregnant women during the COVID-19 pandemic has been related to pregnancy outcomes. Previous studies have shown that anxiety and depression during pregnancy are associated with many complications such as preterm birth [39], fetal growth restriction [15,40], postpartum complications [41], hypertension, preeclampsia, and gestational diabetes [42]. In this study, no correlation was found between the abnormal psychological status of pregnant women and pregnancy outcomes. The results suggest that anxiety and depression symptoms had no significant effect on the incidence of preterm birth, postpartum hemorrhage, and macrosomia (*p* > 0.05). The difference in results may be related to the lack of further stratified analysis of other high-risk obstetric factors in pregnant women.

### Strengths and Limitations

Most of the existing studies have only focused on the changes in psychological status of pregnant women during the COVID-19 pandemic. Our study not only investigated the psychological and sleep status of pregnant women before delivery, but also explored the relationship between psychological status and pregnancy outcomes. A limitation of this study is that it was a single-center study, which may restrict its generalizability. Another limitation is that the gold standard for the diagnosis of psychological problems remains structured clinical interviews, and because of the epidemic, the assessment of anxiety, depression and sleep quality could only be completed through online questionnaires; detailed face-to-face interviews were not possible. Therefore, in this study, we only evaluated whether pregnant women had anxiety or depressive symptoms based on the scores of scales and did not use the diagnosis of anxiety or depression.

## 5. Conclusions

The COVID-19 outbreak has had a significant impact on the mental health of pregnant women and increased the risk of anxiety and depression symptoms. Therefore, regardless of society, hospitals, or families, more attention should be paid to the mental health of pregnant women. There has been an urgent need for social policy intervention and collaboration between obstetricians, family members and even psychologists during the outbreak period of the COVID-19 pandemic. Long-term family companionship should be provided as much as possible to prevent pregnant women from paying too much attention to pandemic-related information. In addition, timely and effective lifestyle guidance and psychological support should also be provided for pregnant women, so as to reduce the adverse psychological effects of COVID-19.

## Figures and Tables

**Table 1:** Distribution of anxiety, depression symptoms and sleep disorders in pregnant women during the COVID-19 pandemic (%).

Characteristics		Total Sample (*n* = 986)
SAS level, No. (%)	No anxiety symptoms	854 (86.6)
	Mild anxiety	112 (11.4)
	Moderate anxiety	15 (1.5)
	Severe anxiety	5 (0.5)
EPDS level, No. (%)	Low depression risk	806 (81.7)
	Moderate depression	104 (10.5)
	Severe depression	76 (7.8)
PSQI level, No. (%)	No sleep disorder	624 (63.3)
	With sleep disorder	362 (36.7)

**Table 2:** Risk factors for the psychological status of pregnant women during the COVID-19 pandemic.

Variables	Anxiety Symptoms No. (%)			Depression Symptoms No. (%)		
	No	Yes	*p* Value	Mild	Moderate to Severe	*p* Value
Age			0.907			0.365
<35	779 (79.0)	120 (12.2)		738 (74.8)	161 (16.3)	
≥35	75 (7.6)	12 (1.2)		68 (6.9)	19 (1.9)	
Residence			0.602			0.984
Urban	458 (46.5)	74 (7.5)		435 (44.1)	97 (9.8)	
Rural	396 (40.2)	58 (5.9)		371 (37.6)	83 (8.4)	
Employment			0.925			0.578
Employed	804 (81.5)	124 (12.6)		757 (76.8)	171 (17.3)	
Unemployed	50 (5.1)	8 (0.8)		49 (5.0)	9 (0.9)	
Income			0579			0.586
<10 w	147 (14.9)	23 (2.3)		137 (13.9)	33 (3.3)	
10–30 w	477 (48.4)	79 (8.0)		451 (45.7)	105 (10.6)	
≥30 w	230 (23.3)	30 (3.0)		218 (22.1)	42 (4.3)	
Education			0.596			0.172
High school or less	111 (11.3)	17 (1.7)		112 (11.4)	16 (1.6)	
Bachelor	604 (61.3)	98 (9.9)		570 (57.8)	132 (13.4)	
Master or higher	139 (14.1)	17 (1.7)		124 (12.6)	32 (3.2)	
Gestational week			0.197			0.038
13–28 w	110 (11.2)	10 (1.0)		108 (11.0)	12 (1.2)	
<13 w	371 (37.6)	58 (5.9)		349 (35.4)	80 (8.1)	
≥28 w	373 (37.8)	64 (6.5)		349 (35.4)	88 (8.9)	
Gravidity			0.472			0.945
1	462 (47.0)	76 (7.7)		440 (44.7)	98 (10.0)	
≥2	390 (39.6)	56 (5.7)		364 (37.0)	82 (8.3)	
Parity			0.375			0.521
1	647 (65.8)	105 (10.7)		611 (62.2)	141 (14.3)	
≥2	204 (20.8)	27 (2.7)		192 (19.5)	39 (4.0)	
Number of fetuses			0.472			0.945
1	462 (47.0)	76 (7.7)		440 (44.7)	98 (10.0)	
≥2	390 (39.6)	56 (5.7)		364 (37.0)	82 (8.3)	
Hypertension			0.217			0.304
No	779 (79)	116 (11.8)		728 (73.8)	167 (16.9)	
Yes	75 (7.6)	16 (1.8)		78 (7.9)	13 (1.3)	
FGR			0.298			0.568
No	803 (81.4)	121 (12.3)		757 (76.8)	167 (16.9)	
Yes	51 (5.2)	11 (1.1)		49 (5.0)	13 (1.3)	
Abnormal amniotic fluid			0.458			0.418
No	826 (83.8)	126 (12.8)		780 (79.1)	172 (17.4)	
Yes	28 (2.8)	6 (0.6)		26 (2.6)	8 (0.8%)	
Being quarantined			0.474			0.762
No	360 (36.5)	60 (6.1)		342 (34.7)	78 (7.9)	
Yes	494 (50.1)	72 (7.3)		464 (47.1)	102 (10.3)	
Sleep disorder			0.000			0.000
No	580 (58.8)	44 (4.5)		558 (56.6)	66 (6.7)	
Yes	274 (27.8)	132 (13.4)		248 (25.2)	114 (11.6)	
Epidemic attention time			0.034			0.955
<1 h/day	602 (61.1)	81 (8.2)		558 (56.6)	125 (12.7)	
≥1 h/day	252 (25.6)	51 (5.2)		248 (25.2)	55 (5.6)	
Time with spouse			0.031			0.022
Constant/decreasing	305 (30.9)	60 (6.1)		286 (29.0)	80 (8.1)	
Increasing	549 (55.7)	72 (7.3)		520 (52.7)	100 (10.1)	

**Table 3:** Logistic regression analysis of risk factors for anxiety and depression in pregnant women during COVID-19.

		OR (95% CI)	*p* Value
Anxiety			
	Epidemic attention time		0.027
	<1 h/d	1	
	≥1 h/d	1.568 (1.052–2.338)	
	Sleep disorders		0.000
	No	1	
	Yes	4.166 (2.797–6.205)	
	Time with spouse		0.035
	Constant/decreasing	1	
	Increasing	0.629 (0.409–0.967)	
Depression			0.048
	Educational level		
	High school or less	1	
	Bachelor or above	1.833 (1.004–3.345)	
	Sleep disorders		0.000
	No	1	
	Yes	3.839 (2.718–5.432)	

**Table 4:** The relationship among anxiety, depressive symptoms, sleep status and pregnancy outcomes.

	Preterm Birth No. (%)			Postpartum Hemorrhage No. (%)			Macrosomia No. (%)		
(*N*/%)	No	Yes	*p* Value	No	Yes	*p* Value	No	Yes	*p* Value
Anxiety			0.285			0.442			0.557
No	822 (83.4)	32 (3.2)		837 (84.9)	17 (1.7)		821 (83.3)	33 (3.3)	
Mild	106 (10.8)	6 (0.6)		108 (11.0)	4 (0.4)		109 (11.1)	3 (0.3)	
≥Moderate	18 (1.8)	2 (0.2)		20 (2.0)	0 (0.0)		20 (2.0)	0 (0.0)	
Depression			0.444			0.210			0.285
Mild	774 (78.5)	32 (3.2)		789 (80.0)	17 (1.7)		773 (78.4)	33 (3.3)	
Moderate	101 (10.2)	3 (0.3)		100 (10.1)	4 (0.4)		102 (10.3)	2 (0.2)	
Severe	71 (7.2)	5 (0.5)		76 (7.7)	0 (0.0)		75 (7.6)	1 (0.1)	

## Data Availability

The datasets used and/or analyzed during the current study are available from the corresponding author upon reasonable request.
